# Multifocal Small Bowel Gastrointestinal Stromal Tumors and Concurrent Neuroendocrine Tumor in Neurofibromatosis Type 1

**DOI:** 10.14309/crj.0000000000002156

**Published:** 2026-06-02

**Authors:** Bianca Thakkar, Teresa Da Cunha, Peter Zanchelli, Murali Dharan

**Affiliations:** 1 Department of Medicine, UConn John Dempsey Hospital, Farmington, CT; 2 Division of Gastroenterology and Hepatology, Department of Medicine, University of Connecticut, Farmington, CT; 3 Department of Pathology and Laboratory Medicine, Hartford Hospital, Hartford, CT

**Keywords:** neurofibromatosis, GIST, neuroendocrine tumor

## Abstract

Neurofibromatosis type I (NF1) is associated with a distinct subset of gastrointestinal stromal tumors (GISTs) but is only rarely linked to neuroendocrine tumors. We describe a 61-year-old woman with NF1 who developed multifocal jejunal GISTs with a synchronous duodenal neuroendocrine tumor (an exceptionally uncommon coexistence). NF1-associated GISTs are typically multifocal, small-bowel predominant, and poorly responsive to targeted medical therapy, rendering surgical resection the primary treatment strategy. This case highlights the unique tumor biology of NF1 and emphasizes the importance of meticulous anatomic evaluation and coordinated multidisciplinary surgical planning to determine the optimal extent and approach to resection.

## INTRODUCTION

Neurofibromatosis type I (NF1) is a genetic syndrome characterized by mutations in the NF1 tumor suppressor gene, often leading to the development of multiple benign and malignant neoplasms. Among these, gastrointestinal stromal tumors (GISTs) represent a rare but notable manifestation. Unlike sporadic GISTs, those associated with NF1 typically present as multifocal lesions, predominantly in the small bowel. NF1-associated GISTs are typically wild-type at the molecular level for activating mutations in the *KIT* and platelet-derived growth factor receptor alpha (*PDGFRA*) genes, although they frequently express c-KIT (CD117) protein on immunohistochemistry (IHC), an important distinction with diagnostic and therapeutic implications.^1,2^

## CASE REPORT

A 61-year-old woman with a history of NF1, hypertension, hyperlipidemia, gastroesophageal reflux disease, and hypothyroidism presented with chronic abdominal pain. Computed tomography (CT) imaging revealed multiple nonobstructing small bowel masses, including jejunal lesions measuring 3.1, 1.6, and 0.6 cm, as well as a possible 1.2 cm duodenal lesion and a 2.6 × 1.7 cm circumscribed lesion at the D2/uncinate process, initially suggestive of a pancreatic vs duodenal lipoma.

The patient was discharged with outpatient follow up re-presented to the emergency department 1 week later with worsening abdominal pain and decreased oral intake. In the absence of obstruction, symptoms were attributed to bulky small bowel lesions, and she underwent exploratory laparotomy with resection of 33 cm of jejunum containing 3 palpable masses. Pathology revealed 4 jejunal tumors (2.8, 1.3, 0.5, 0.2 cm) composed of spindle cells with a mitotic rate of 5/5 mm^2^, histologic grade G1, and no necrosis. Margins were negative. IHC showed CD117 positivity and negativity for S100, actin, and desmin; DOG1, CD34, Ki-67, and molecular testing were not performed.

Two months later, she underwent endoscopic ultrasound for evaluation of the residual duodenal lesion previously seen on CT. An intramural mass at the D2/uncinate process arising from the muscularis propria (MP) was identified, with differential including neuroendocrine tumor (NET) vs stromal lesion. Fine needle aspiration demonstrated neoplastic cells with mild atypia and coarse chromatin; immunostaining was positive for chromogranin and synaptophysin, consistent with a NET. Ki-67 was not reported. Magnetic resonance imaging (MRI) showed a 0.8 cm enhancing lesion at the D2/D3 junction consistent with the previously observed NET on endoscopic ultrasound. ^64^Cu-DOTATATE positron emission tomography/CT showed no radiotracer uptake and no metastatic disease. Endoscopic resection attempted 6 months later was unsuccessful due to poor margin visualization, MP involvement, and proximity to the pancreas. Repeat positron emission tomography/CT again demonstrated a 0.9 × 0.7 cm lesion without radiotracer uptake.

Given these findings, along with the known risk of nodal metastasis even in small duodenal NETs, a pylorus-sparing pancreaticoduodenectomy with regional lymphadenectomy was pursued to achieve complete oncologic resection and appropriate nodal staging. The procedure required extensive adhesiolysis but was completed without intraoperative complications (estimated blood loss 200 mL). No evidence of metastatic disease was noted.

**Figure 1.**
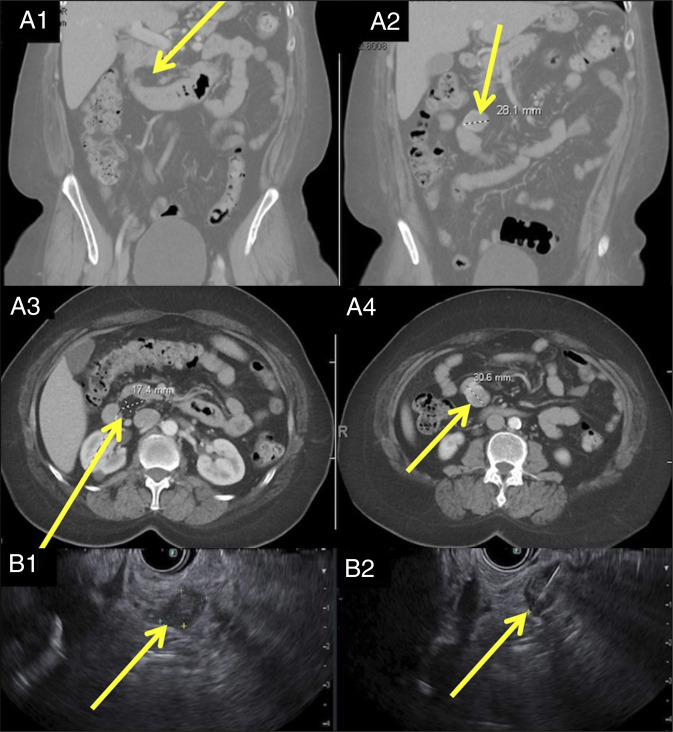
Cross-sectional imaging and endoscopic ultrasound. Computed tomography abdomen/pelvis with several nonobstructing small bowel masses visualized, concerning for gastrointestinal stromal tumors associated with neurofibromatosis 1 (A1–A4). Endoscopic ultrasound with a mucosal lesion (B1) in the duodenum and an intramural (subepithelial) lesion (B2) in the duodenum that appeared to originate from within the muscularis propria.

**Figure 2.**
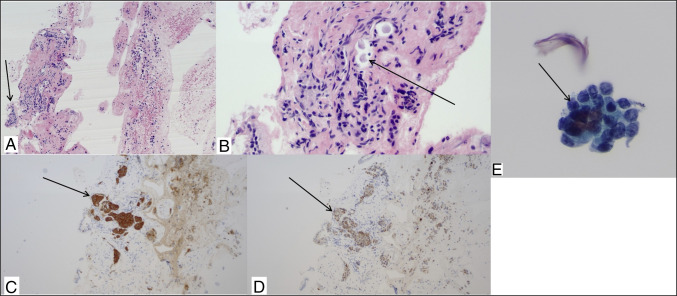
Histopathology. Duodenum, fine needle aspirate, cell block shows mucosa with adjacent nested cells forming a mass (a, 100×), and on higher magnification of the cells shows monotonous bland polygonal cells with coarse chromatin (B, 400×). Immunohistochemical staining, the nested proliferation of polygonal cells both stain positive for synaptophysin (C, 100×) and chromogranin (D, 100×). Duodenum, fine needle aspirate, cytology shows nested cluster of neoplastic cells with mild nuclear atypia, inconspicuous nucleoli and coarse chromatin (E, 400×).

Pathology revealed a 1.1 × 1.0 cm well-differentiated grade 1 duodenal NET invading peripancreatic adipose tissue (pT3N1), with Ki-67 < 3% and <2 mitoses per 2 mm^2^; margins were negative. IHC was positive for AE1/AE3, chromogranin, and synaptophysin, and negative for desmin and CD117. In addition, a 0.3 cm grade 1 GIST (pT1N0) with Ki-67 <3% and ≤1 mitosis per 5 mm^2^ was identified, with negative margins; IHC was positive for CD117 and negative for desmin, AE1/AE3, chromogranin, and synaptophysin. The NET was classified as stage III (pT3N1), indicating higher recurrence risk despite low proliferative activity, while the GIST was very low risk per Armed Forces Institute of Pathology criteria. Twelve lymph nodes were examined, with 3 positive for NET and none for GIST (Figures 1 and 2).

The postoperative course was complicated by pancreatic fistula requiring prolonged drainage and total parenteral nutrition, delayed gastric emptying with ileus, pulmonary embolism, portal vein thrombosis, hemorrhagic complications requiring endovascular intervention, hemoperitoneum, and recurrent right pleural effusions requiring thoracenteses, resulting in a prolonged hospitalization of approximately 2 months.

Following discharge, her clinical course was notable for failure to thrive despite pancreatic enzyme replacement therapy and nutritional support. The patient was readmitted and is currently undergoing inpatient work up for elevated liver enzymes of unclear etiology, although multifactorial causation is suspected.

## DISCUSSION

NF1 is an autosomal dominant disorder characterized by cutaneous neurofibromas, café-au-lait macules, and a predisposition to various neoplasms, including GISTs.^1,3^ NF1 is diagnosed when ≥2 characteristic clinical features are present (or ≥1 feature if a parent is affected), including café-au-lait macules, neurofibromas, axillary/inguinal freckling, optic pathway glioma, Lisch nodules or choroidal abnormalities, distinctive osseous lesions, or a pathogenic NF1 gene variant.^4^ Of note, our patient had a presumed clinical diagnosis of NF1 since the age of 10 based on a strong family history (grandparent, parent, sibling, and 2 children with clinically diagnosed NF1) and clinical symptoms (numerous cutaneous neurofibromas and cafe au lait spots, optic glioma). Genetic testing was obtained to allow targeted testing for her children and other family members, which confirmed a 17q11.2 deletion consistent with NF1.

The co-occurrence of GIST and NET in NF1 is extremely rare and poses diagnostic and management challenges. Duodenal NETs are reported in only about 1% of individuals with NF1 with a prevalence of 1 in 40 million people.^5,6^ NETs are a type of carcinoid tumor that are typically malignant and originate from endocrine cells within the gastrointestinal tract and most commonly present in the periampullary region.^5,6^ Therefore, patients typically present with jaundice and vague abdominal pain.^5^

NF1-GISTs are clinically and genetically distinct from sporadic GISTs.^7^ NF1-GISTs most commonly arise in the small intestine (jejunum and ileum), with multifocality observed in up to 54% of cases, whereas sporadic GISTs most commonly arise in the stomach.^8,9^ The median age at diagnosis is younger than sporadic GISTs (median 49 vs 59 years), and there is no clear sex predilection.^1^ NF1-GISTs are often associated with cutaneous stigmata of NF1, such as café-au-lait macules and neurofibromas and the most common presenting symptoms are gastrointestinal bleeding, obstruction, or perforation.^5^ Many cases are discovered incidentally or after years of intermittent bleeding.^10,11^

Diagnostic workup should include endoscopy, cross-sectional imaging (CT/MRI), and biopsy of suspicious lesions. Imaging modalities such as multiphasic CT and endoscopic evaluation are essential for localization and characterization.^12,13^ Histologically, NF1-GISTs are predominantly spindle cell tumors, often with low mitotic activity and skeinoid fibers.^1,14^ IHC typically demonstrates strong CD117 positivity, with frequent CD34 and occasional S-100 expression.^1,14^ Hyperplasia of the interstitial cells of Cajal is commonly observed in adjacent tissue.^10,12,14^ Both the American Society of Clinical Oncology and National Comprehensive Cancer Network (NCCN) guidelines recommend *KIT* and *PDGFRA* mutational analysis in patients for whom systemic therapy is being considered, as mutation status predicts response to TKIs.^15,16^

A key limitation of this case is the absence of molecular testing for *KIT* and *PDGFRA* mutations. NF1-associated GISTs are typically molecularly wild-type, lacking activating *KIT/PDGFRA* mutations, and instead arise from somatic inactivation of the NF1 allele with downstream mitogen activated protein kinase pathway activation.^7^ This distinction has direct therapeutic implications, as NF1—GISTs are driven by loss of neurofibromin rather than constitutive tyrosine kinase signaling and therefore demonstrate limited responsiveness to imatinib.^17^

In tumors lacking *KIT/PDGFRA* mutations, further molecular evaluation, particularly for succinate dehydrogenase deficiency, is recommended to clarify classification and guide genetic counseling.^15,16^ Although molecular testing was not performed in this case, the diagnosis of NF1-associated GIST is strongly supported by the clinical context of NF1, multifocal small bowel involvement, and characteristic histopathologic features. However, incorporation of molecular profiling would enhance diagnostic precision and may have implications for prognostication and eligibility for targeted therapies or clinical trials.

Management is further complicated by the coexistence of a NET. In NF1, NETs most commonly arise in the periampullary region but may occur throughout the gastrointestinal tract, warranting evaluation with appropriate biochemical markers and staging imaging.^18,19^ Therapeutically, the role of neoadjuvant or adjuvant imatinib is limited in NF1-GIST due to the absence of targetable *KIT/PDGFRA* mutations.^7,17^ Consequently, surgical resection remains the primary treatment modality. However, unlike hereditary diffuse gastric cancer (*CDH1* carriers), no standard guidelines exist for the timing or extent of surgery in germline or NF1-associated GISTs.^9^

Management decisions must therefore balance the generally indolent course of these tumors against the morbidity of surgical intervention.^9^ Germline GISTs are typically initially observed due to their indolent growth^9^ with elective resection typically reserved for symptomatic patients, for tumors with accelerated growth, or tumors near a vital structure (i.e., pancreas or superior mesenteric artery) where future resectability may be compromised.^9^ In cases of multifocal disease, segmental or partial small bowel resections are typically employed to achieve oncologic control while preserving bowel length.^8,20^ Emergent surgery is rarely indicated unless there is concern for bleeding, intestinal perforation, or tumor rupture.^9^

For NF1 patients with multifocal GISTs and NETs, simultaneous resection of both GIST and NET may be required, necessitating careful preoperative localization and staging.^18,21,22^ According to the NCCN guidelines, endoscopic resection or transduodenal local excision with or without lymph node sampling and pancreatoduodenectomy are options recommended for localized nonmetastatic duodenal NETs,^23^ with the choice guided by tumor size, location, and nodal involvement.^19,24^ For patients presenting with NETs in the jejunum, ileum, or colon, surgical resection of the bowel with regional lymphadenectomy is recommended.^23^ Complete resection with negative margins is the goal and regional lymph node sampling is essential for NETs due to their propensity for nodal metastasis.^19,24^

In our case, management required multidisciplinary discussion between gastroenterology and surgical oncology regarding the optimal approach to resection of the NET. MRI was obtained to better delineate tumor origin, as initial CT was inconclusive for duodenal vs pancreatic uncinate involvement. MRI confirmed a duodenal lesion. Although primary, nonmetastatic duodenal NETs are typically amenable to endoscopic resection, this was not feasible due to MP involvement and the location at the D2-D3 junction, which limited margin visualization and safe complete resection. Therefore, definitive surgical resection with pancreaticoduodenectomy was pursued.

The risk of recurrence and mortality after surgical resection is similar between NF1-GIST and sporadic GIST patients. Prognosis is generally favorable for small, low-mitotic-rate tumors; however, tumors >5 cm or with high mitotic activity (>5/50 high power fields) are associated with increased risk of metastasis.^8,11^ In metastatic or unresectable cases, alternative strategies such as MAPK kinase inhibitors are under investigation but are not yet standard of care.^7,11^ For our patient, postresection surveillance should be guided primarily by the NET, as the coexisting GIST is very low risk. Per NCCN guidelines, very low-risk GISTs (<2 cm, low mitotic rate) requires minimal or no routine surveillance.^16^ By contrast, the resected pT3N1 duodenal NET warrants long-term surveillance with cross-sectional imaging every 6–12 months for at least 10 years, with consideration of somatostatin receptor imaging if recurrence is suspected.^25^

This case highlights the importance of recognizing the unique presentation and molecular behavior of NF1-associated GISTs. The coexistence of multifocal GIST and NET further complicates management, underscoring the need for a multidisciplinary approach. More research is needed to develop standardized protocols for this rare clinical scenario.

## DISCLOSURES

Author contributions: B. Thakkar provide the concept of the case report and drafting the work along with acquisition, analysis, or interpretation of data for the work. T. Da Cunha assisted with drafting the work and providing edits. P. Zanchelli acquired data including the pathology images and descriptions and assisted with writing the pathology descriptions. M. Dharan supervised the case and provided critical edits and approved the final work. B. Thakkar is the article guarantor.

Financial disclosure: None to report.

Previous presentation: Presented at the American College of Gastroenterology Annual Scientific Meeting, October 25–29, 2026; Phoenix, AZ.

Informed consent was obtained for this case report.

## ABBREVIATIONS:

CT: computed tomography
GIST: gastrointestinal stromal tumor
IHC: immunohistochemistry
MP: muscularis propria
MRI: magnetic resonance imaging
NET: neuroendocrine tumor
NF1: neurofibromatosis 1
PDGFRA: platelet-derived growth factor receptor alpha
